# Comparison of hemodynamics and root configurations between remodeling and reimplantation methods for valve-sparing aortic root replacement: a pulsatile flow study

**DOI:** 10.1007/s00595-022-02622-4

**Published:** 2022-11-27

**Authors:** Masahiro Seki, Takashi Kunihara, Jyunpei Takada, Kenichi Sasaki, Ryo Kumazawa, Hiroshi Seki, Saeko Sasuga, Hirotsugu Fukuda, Mitsuo Umezu, Kiyotaka Iwasaki

**Affiliations:** 1 Department of Cardiovascular Surgery, Dokkyo Medical University, Mibu, Japan; 2 Department of Cardiac Surgery, Jikei University School of Medicine, Tokyo, Japan; 3 Department of Modern Mechanical Engineering, Graduate School of Creative Science and Engineering, Waseda University, Tokyo, Japan; 4 Department of Cardiovascular Surgery, Saitama Sekishinkai Hospital, Sayama, Japan; 5 Division of Cardiovascular Surgery, Yamato Seiwa Hospital, Yamato, Japan; 6 Department of Integrative Bioscience and Biomedical Engineering, Graduate School of Advanced Science and Engineering, Waseda University, Tokyo, Japan; 7 Cooperative Major in Advanced Biomedical Sciences, Joint Graduate School of Tokyo Women’s Medical University and Waseda University, Waseda University, 2-2 Wakamatsucho, Shinjuku, Tokyo 162-8480 Japan

**Keywords:** Valve-sparing root replacement, Aortic valve repair, Aortic root remodeling, Aortic valve reimplantation, Aortic annuloplasty, Pulsatile flow study

## Abstract

**Purpose:**

To compare the characteristics of reimplantation (RI) using grafts with sinuses and remodeling (RM) with/without external suture annuloplasty using a pulsatile flow simulator.

**Methods:**

Porcine aortic roots were obtained from an abattoir, and six models of RM and RI with sinuses were prepared. External suture annuloplasty (ESA) was performed in the RM models to decrease the root diameter to 22 mm (RM-AP22) and 18 mm (RM-AP18). Valve models were tested at mean pulsatile flow and aortic pressure of 5.0 L/min and 120/80 (100) mmHg, respectively, at 70 beats/min. The forward flow, regurgitation, leakage, backflow rates, valve-closing time, and mean and peak pressure gradient (p-PG) were evaluated. Root configurations were examined using micro-computed tomography (micro-CT).

**Results:**

The backflow rate was larger in the RM models than in the RI models (RI: 8.56% ± 0.38% vs. RM: 12.64% ± 0.79%; *p* < 0.01). The RM-AP and RI models were comparable in terms of the forward flow, regurgitation, backflow rates, p-PG, and valve-closing time. The analysis using a micro-CT showed a larger dilatation of the sinus of the Valsalva in the RM groups than in the RI group (Valsalva: RI, 26.55 ± 0.40 mm vs. RM-AP22, 31.22 ± 0.55 mm [*p* < 0.05]; RM-AP18, 31.05 ± 0.85 mm [*p* < 0.05]).

**Conclusions:**

RM with ESA and RI with neo-sinuses showed comparable hemodynamics. ESA to RM reduced regurgitation.

**Supplementary Information:**

The online version contains supplementary material available at 10.1007/s00595-022-02622-4.

## Introduction

Aortic root remodeling (RM) and aortic valve reimplantation (RI), the two major procedures for valve-sparing root replacement (VSRR), have evolved to show excellent clinical results. The favorable long-term durability of VSRR without the need for life-long anticoagulation therapy has contributed to an improved quality of life in young patients. Nevertheless, the optimal procedure for VSRR remains controversial [1].

RM is considered advantageous because of its physiological hemodynamics and reduced aortic valve systolic energy loss [2]. In contrast, RI is favored for its annulus stability and is chosen especially for patients with annuloaortic ectasia or Marfan syndrome [3–5]. However, the use of the tube graft eliminates the mobility of the sinuses of Valsalva in the RI technique, which leads to rapid and unphysiological valve behavior [6].

Recent reports have shown that RI using a graft with sinuses provides better valve behavior than that using the straight graft due to the preservation of the distensibility of the neo-Valsalva sinus [7]. With regard to RM, concomitant annuloplasty procedures have been reported to preserve the physiological hemodynamics of the valve and annulus stability [3, 8, 9]. However, the valve behaviors in RM with annuloplasty and RI using grafts with sinuses have never been compared in detail.

The present study, therefore, compared the hemodynamics and root configurations of the two modern VSRR techniques in a pulsatile flow simulator to gain insight into the influence of VSRR techniques in clinical practice.

## Methods

### Preparation of valves

**Fig. 1:**
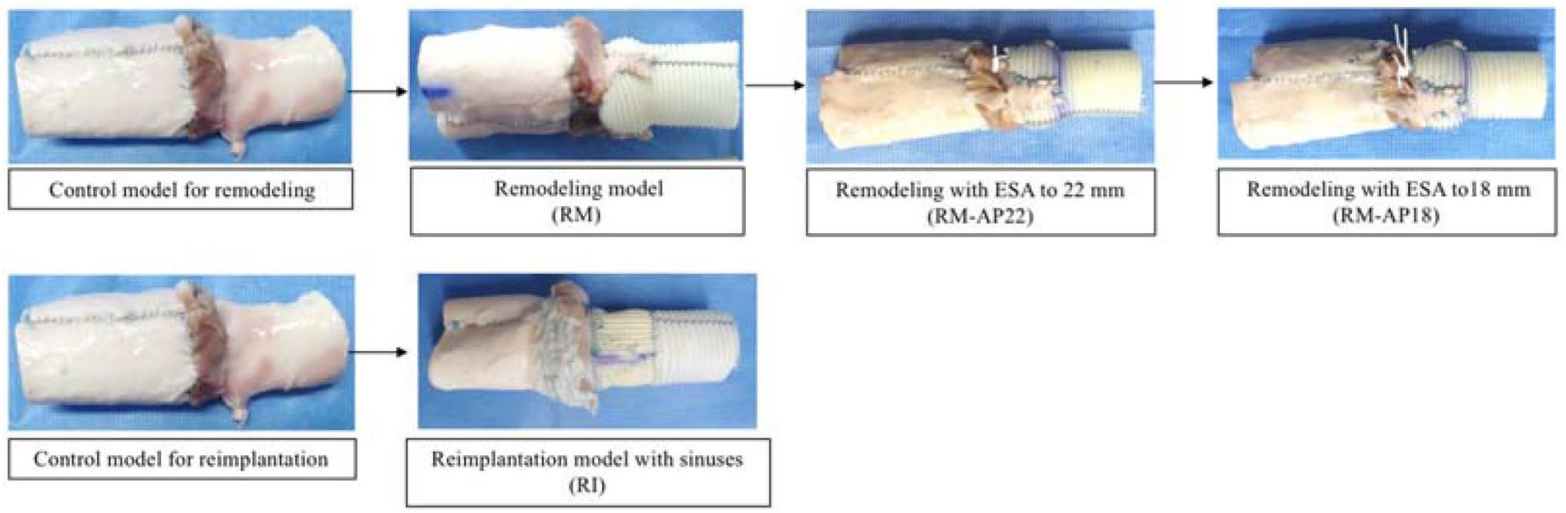
Preparation of the valves. The remodeling and reimplantation models were prepared using porcine aortic roots. A 24-mm Dacron graft was used in both models. Grafts with sinuses were handcrafted in the reimplantation model. External suture annuloplasty was used as a remodeling technique to reduce the annulus to 22 or 18 mm

We prepared six RM and RI models using porcine aortic valves. Fresh porcine hearts were obtained from a local abattoir and stored frozen. The hearts were defrosted on the day of the experiment. The aortic root, including the left ventricular outflow tract (LVOT), was excised. After visual inspection, porcine hearts with undamaged tricuspid aortic valves were used. As a control model, the ascending aorta was cut down and sewn into the remaining muscle of the LVOT using 4–0 or 5–0 synthetic polypropylene sutures to connect the valve models to the pulsatile flow simulator, as shown in Fig. 1. Coronary ostia were ligated using 2–0 silk sutures. Two models were then prepared for each RM and RI technique (*n* = 6 each).

The graft size is decided by the body surface area (BSA) and the normal estimated ventriculo-aortic junction (VAJ) of the patient in clinical practice. As Capps et al. [10] mentioned regarding the correlation between the VAJ and BSA, the appropriate diameter of the VAJ is set to be around 20–22 mm. The ratio between VAJ and sinotubular junction in the normal subject has been reported as 1:1.1 to 1:1.2 [11]. Therefore, a 24-mm tube graft with 20- to 22-mm annuloplasty in the RM models and a 24- to 26-mm Valsalva graft with the RI seemed to be a decent choice in this study. Considering the VAJ of the control group (23.88 mm for the RM and 23.67 mm for the RI), setting the VAJ to 22 mm seemed mild, so a targeted VAJ of 20 mm was decided to be appropriate in this experimental study.

Although the majority of appropriate grafts for RI in clinical practice are 26 mm, considering the use of porcine hearts (smaller BSA than humans) and the targeted VAJ of 20 mm, we chose a smaller graft of 24 mm. To clarify the effect of the annuloplasty procedure, we set the VAJ diameter 4 mm apart (22 to 18 mm) in the RM with annuloplasty groups with the same 24 mm graft size.

For the RM group, a J-graft SHIELD NEO^®^ (Japan Lifeline, Tokyo, Japan) 24-mm tube graft was used. We left at least 5 mm of the aortic wall remnant to include the graft inside the root and secure the anastomosis. The commissure height of the graft was not fixed at a certain value but was tailored to an appropriate commissure height for each porcine model. RM was performed using continuous 5–0 synthetic polypropylene sutures with 3-mm intervals for the native side and 5-mm intervals for the graft to create the bulge of the Valsalva. The RM group underwent external suture annuloplasty (ESA) to decrease the diameter of the VAJ to 22 mm (RM-AP22) or 18 mm (RM-AP18). ESA was performed with the method described by Schneider et al. [8] using expanded polytetrafluoroethylene (e-PTFE: Gore-Tex CV-0; W. L. Gore, Flagstaff, AZ, USA). ESA was performed at the level of the basal ring under intravascular visual guidance. Right/non-commissure suturing was not performed to avoid membranous septum interference. The suture was tied down after insertion of 22- or 18-mm Hegar dilators (MA Corporation, Chiba, Japan, distributed by JP Creed Corporation, Tokyo, Japan) into the aortic annulus.

For the RI model, considering the effects of the sinus of Valsalva on the physiological valve motion [6, 7, 12, 13], Dacron grafts with neo-sinuses were handmade by combining the horizontal and vertical creases of the grafts. The direction of the groove at the collar and straight portion is horizontal, whereas that at the sinus of Valsalva is vertical. The handmade neo-sinus Valsalva graft was crafted based on the proportion of the Gelweave™ Valsalva (Terumo Vascutek, Tokyo, Japan). A J-graft SHIELD NEO® with a 24-mm diameter was used for this purpose. In reference to the product information and observation of the actual item, three rectangle grafts were crafted for the recreation of the sinuses. The rectangle grafts were combined to leave about 10 vertical creases in width and 24 mm in length for each sinus. This vertical groove area served as the neo-sinus of Valsalva. The horizontal area was left on the proximal side of the graft as a collar with the length of three creases (basal ring). First-row suturing was performed with pledgeted 2–0 braided polyester sutures in a horizontal mattress fashion at the basal ring. A total of six stitches (three below the commissure and three in the nadir) were applied. Second-row suturing was performed with continuous 5–0 polypropylene sutures using the standard method [14]. Commissures were fixed to the border of the Valsalva graft. Fibrin glue (Beri-plast^®^ P; CSL Behring, Marburg, Germany) was used to avoid leakage from the suture line in all models.

### Experimental procedure

For the remodeling experiments, the RM models (*n* = 6) were initially prepared. After testing the hydrodynamic performance of the RM model, ESA was applied to the RM model to prepare the RM-AP22 model. After testing the hydrodynamic performance of the RM-AP22 model, RM-AP18 models were sequentially prepared and tested. The RI models were independently prepared (*n* = 6) and tested.

### Pulsatile flow study

**Fig. 2:**
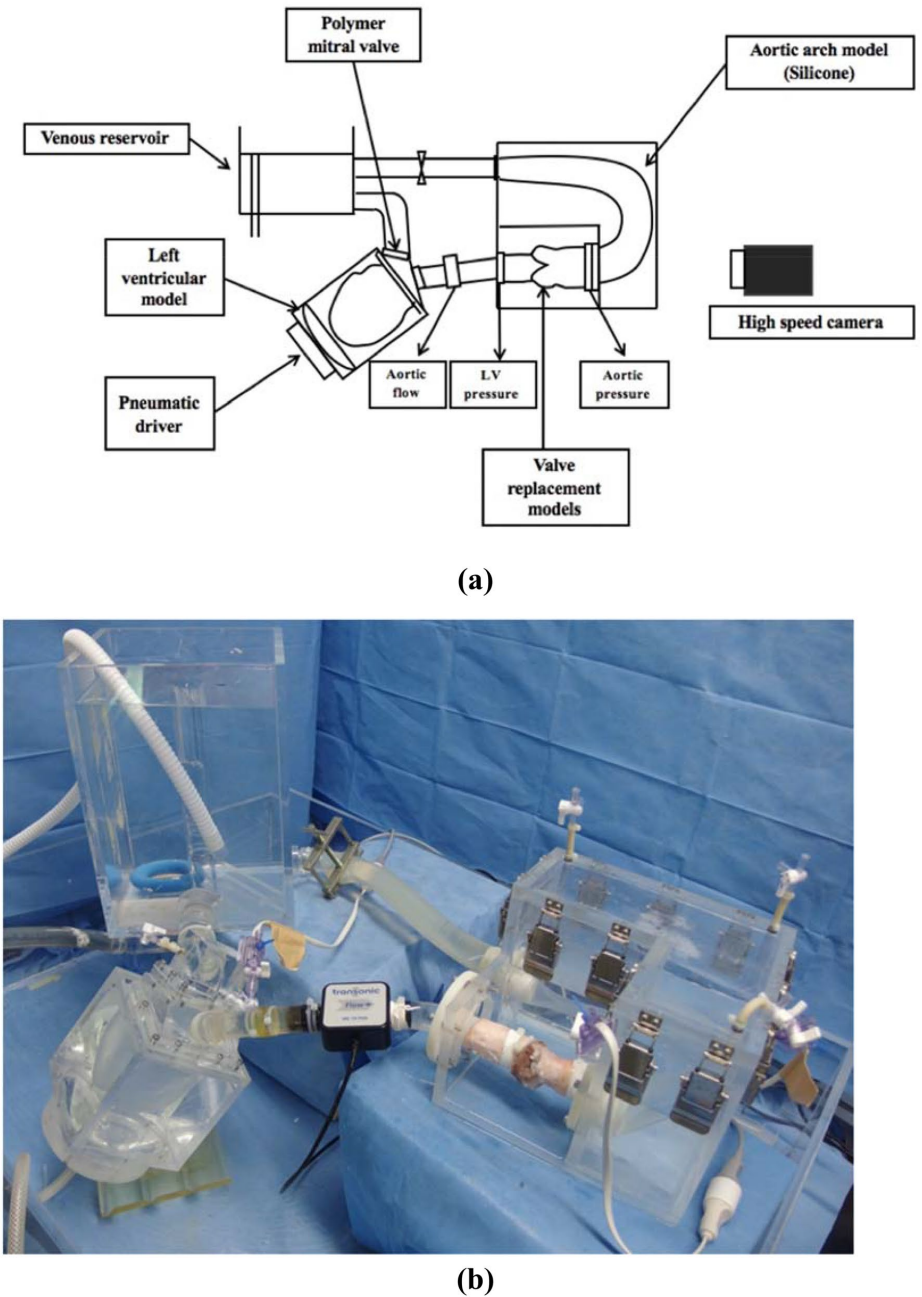
Pulsatile flow simulator. **a** Schematic of the pulsatile flow circuit. **b** An overall view of the pulsatile flow circuit

The influences of the VSRR techniques on valve behaviors were investigated using a pulsatile flow simulator (Fig. 2a, b). Using a porcine aortic valve before conducting any VSRR procedures (control model), the mean pulsatile flow rate and aortic pressure were regulated to 5 L/min and 120/80 (100) mmHg, respectively. The heart rate was set at 70 beats/min. The RM, RM-AP22, RM-AP18, and RI models were then tested.

**Fig. 3:**
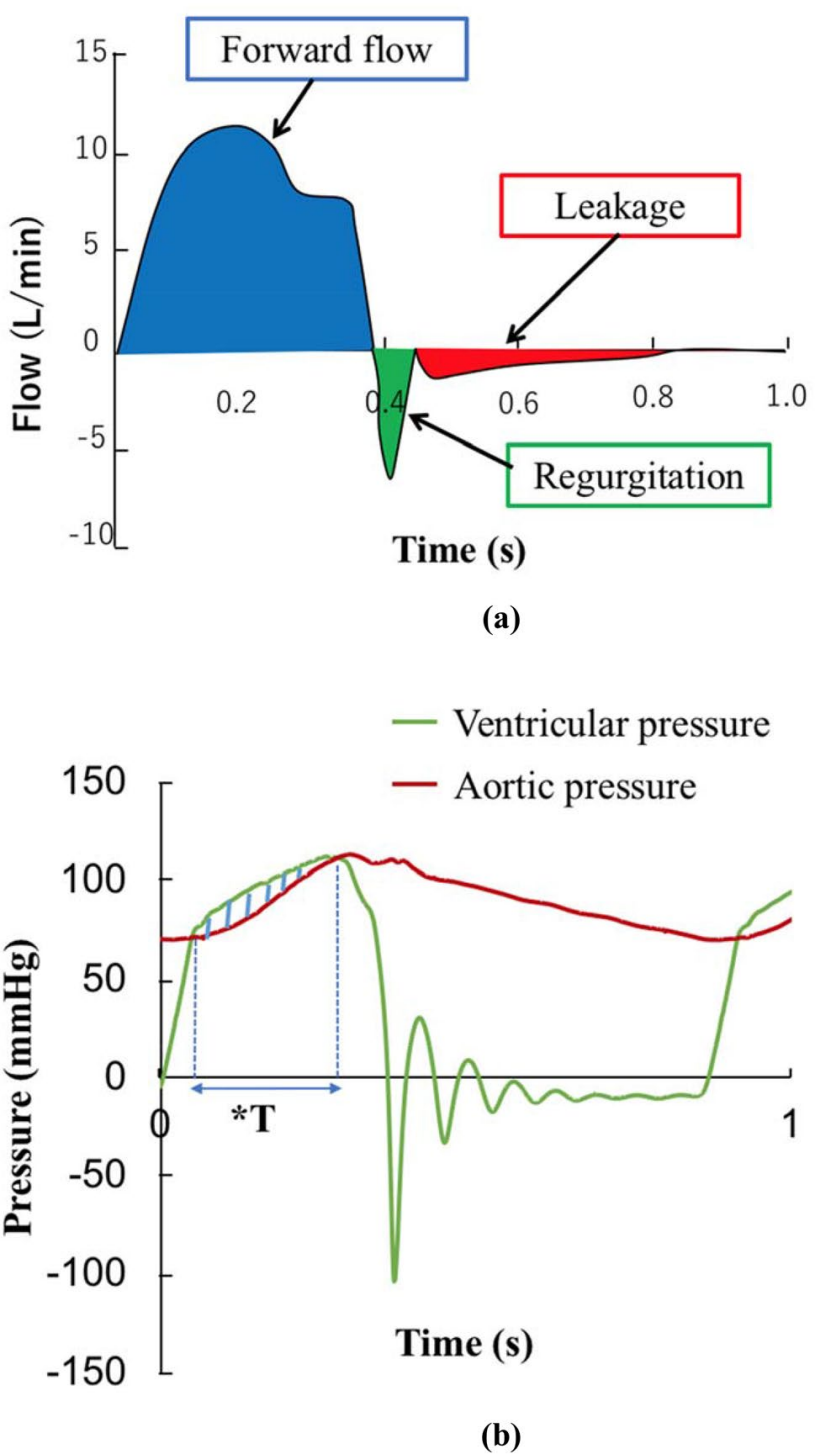
Flow and pressure waveforms. **a** Schematic of pulsatile flow waveforms. Forward flow, regurgitation, and leakage were measured. **b** Pressure waveform. Diagonal lines represent the pressure gradient between the ventricle and aorta. *T = valve opening time

**Fig. 4:**
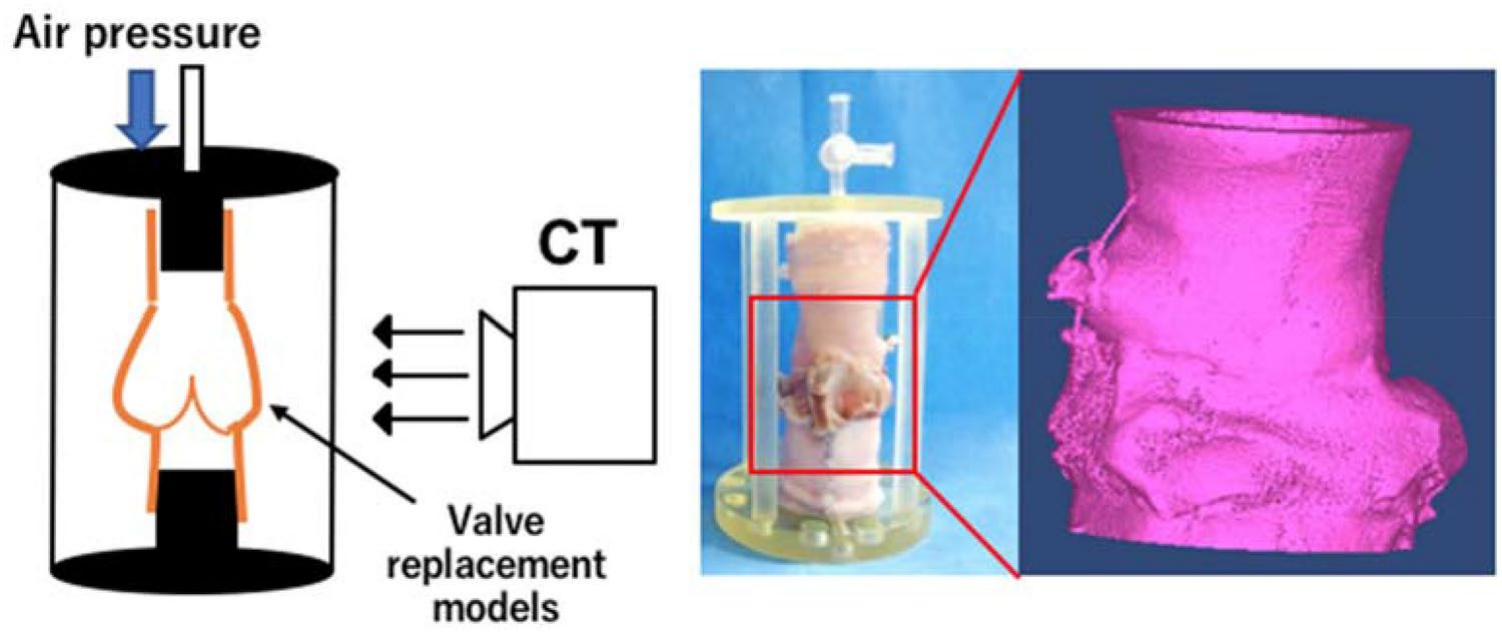
An analysis of the three-dimensional morphological structure of the valves using micro-CT

The flow was measured using an ultrasonic flow sensor (ME-PXN ME19PXN325; Transonic, Ithaca, NY, USA). Left ventricular and aortic pressures were measured using pressure transducers (PXMK10200; Edwards Lifesciences, Irvine, CA, USA). The mean forward flow, regurgitation, leakage, backflow rates, mean pressure gradient (m-PG), and peak pressure gradient (p-PG) were compared among the VSRR models. The mean forward flow rate was measured as the antegrade left ventricular flow rate toward the aortic valves (Fig. 3a). Regurgitation and leakage rates were determined by evaluating retrograde flows during and after closure of the aortic valves (Fig. 3a). The backflow rate was calculated based on the following formula: backflow rate (%) = [(regurgitation + leakage) / mean forward flow rate] × 100. The pressure gradient (PG) of the aortic valve was calculated as the pressure difference between the left ventricular and aortic pressure (Fig. 3b). p-PG was the largest value, whereas m-PG was defined as $\frac{1}{T}\int \Delta P\mathrm{d}t$ (*T* = valve opening time). The valve-closing time was assessed using a high-speed camera at a capture speed of 1000 fps (Keyence Co. Ltd., Osaka, Japan). After each pulsatile flow test, the three-dimensional conduit morphology of each model was analyzed using micro-computed tomography (micro-CT) (Yamato Scientific Co. Ltd., Tokyo, Japan) at a resolution of 91.5 × 91.5 × 91.5 µm^3^. An air pressure of 80 mmHg was applied to the lumen at the aortic side of the models to simulate the pressure conditions during valve closure (Fig. 4). The perimeters of the sinotubular junction (STJ), sinus of Valsalva (Valsalva), and VAJ were measured, and the diameters were calculated.

### Statistical analyses

The Shapiro–Wilk test was used to test the normality of continuous variables. Depending on whether the distribution was normal, a one-way analysis of variance or Kruskal–Wallis test was used to compare the means of the four groups. When there was a significant difference, Tukey’s HSD test or Dunn’s test was used to evaluate the difference in the means of each group as a post hoc analysis. Data are expressed as the mean ± standard error.

The Statistical Package for Social Science (SPSS) version 28 (IBM Corp., Armonk, NY, USA) was used for the analyses. Statistical significance was set at *p* < 0.05.

## Results

### Hemodynamic parameters

**Fig. 5:**
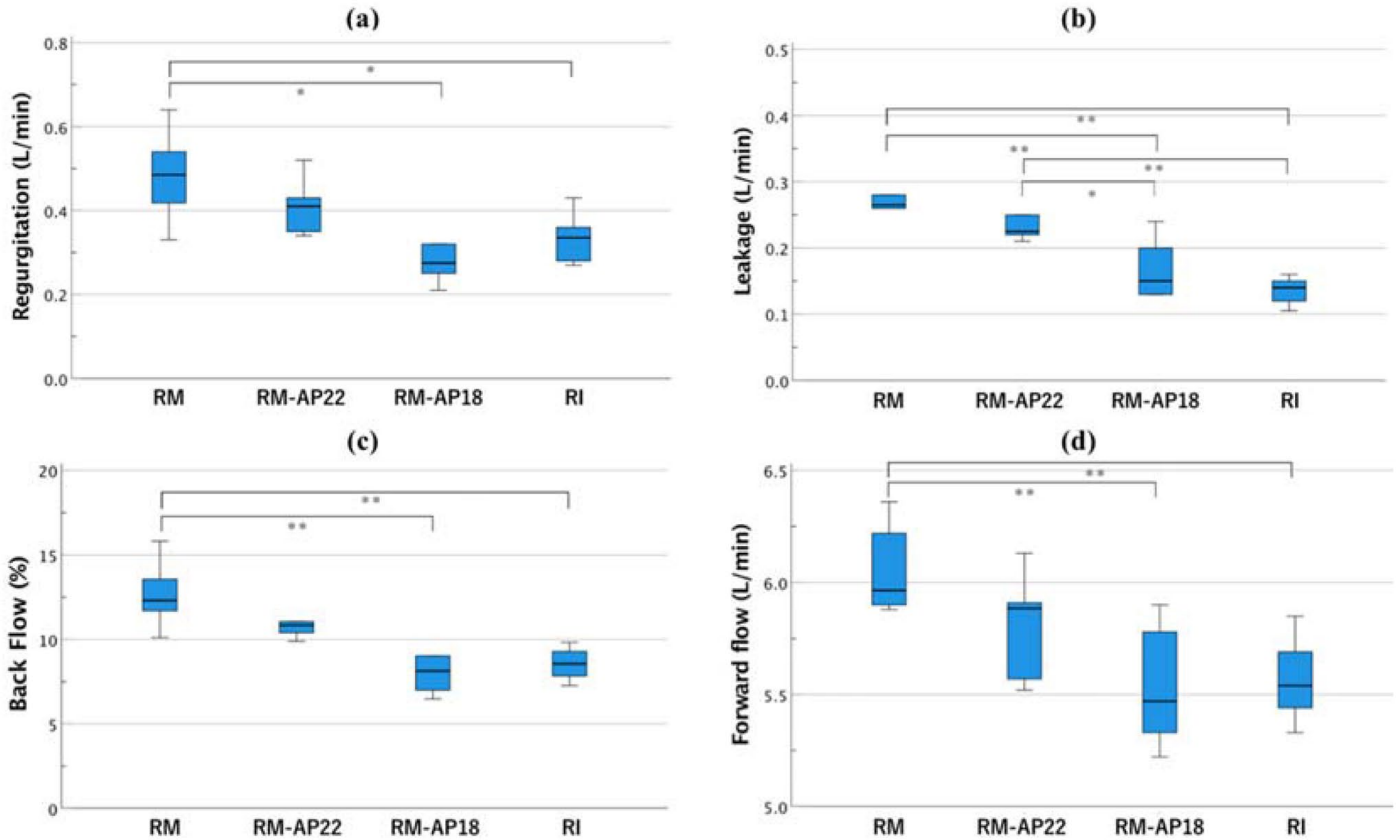
The comparison of hemodynamics. (**a**) Regurgitation flow, (**b**) leakage flow, (**c**) backflow, (**d**) forward flow. *P < 0.05; **P < 0.01

**Fig. 6:**
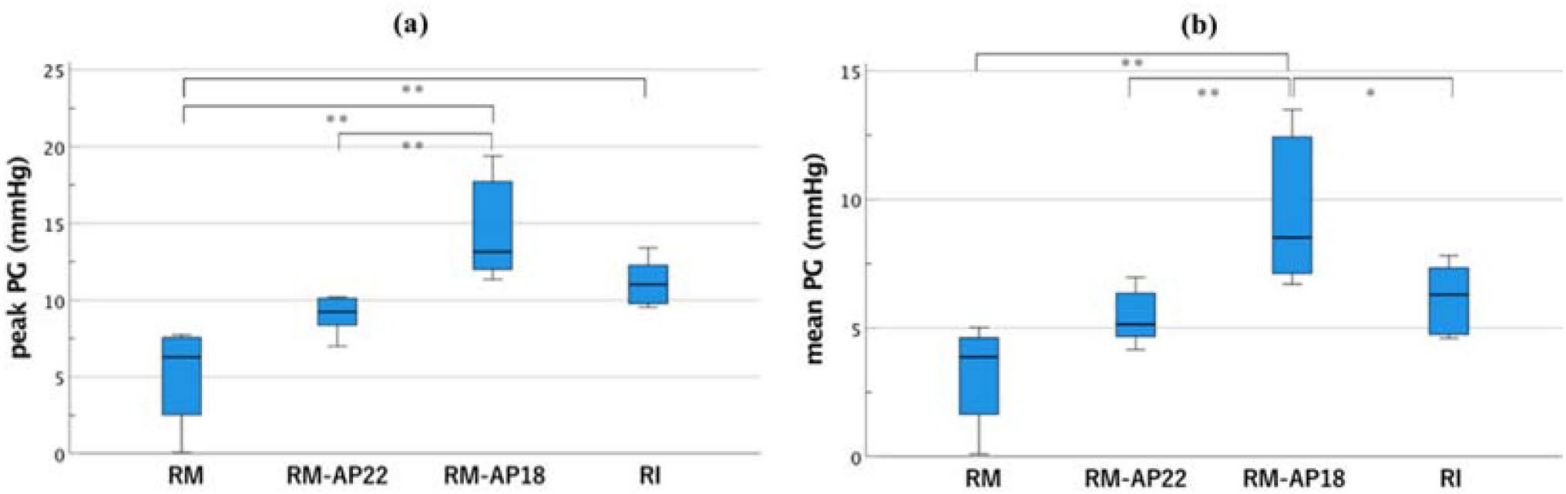
The comparison of pressure gradients during valve opening. (**a**) Peak pressure gradient, (**b**) mean pressure gradient. *P < 0.05; **P < 0.01

- Comparisons among remodeling groups
  Regurgitation, leakage, and backflow rates were lower in RM models with ESA than RM models without ESA (regurgitation: RM, 0.48 ± 0.04 L/min vs. RM-AP18, 0.31 ± 0.05 L/min [*p* < 0.05] and RM-AP22, 0.41 ± 0.07 L/min [*p* = 0.50]; leakage: RM, 0.28 ± 0.02 L/min vs. RM-AP18, 0.17 ± 0.02 L/min [*p* < 0.01] and RM-AP22, 0.23 ± 0.02 L/min [*p* = 0.18]; backflow: RM, 12.64% ± 0.79% vs. RM-AP18, 8.54% ± 0.89% [*p* < 0.01] and RM-AP22, 11.01% ± 0.43% [*p* = 0.33]) (Fig. 5a–c). The forward flow rate was also lower in RM models with ESA than RM models without ESA (RM: 6.05 ± 0.08 L/min vs. RM-AP18: 5.53 ± 0.11 L/min [*p* < 0.01] and RM-AP22: 5.82 ± 0.09 L/min [*p* = 0.30]) (Fig. 5d). RM-AP18 showed a significantly greater PG than RM and RM-AP22 (p-PG: RM-AP18, 14.5 ± 1.3 mmHg vs. RM, 5.1 ± 1.3 mmHg [*p* < 0.01] and RM-AP22, 9.0 ± 0.5 mmHg [*p* < 0.01]; m-PG: RM-AP18, 9.5 ± 1.2 mmHg vs. RM, 3.2 ± 0.8 mmHg [*p* < 0.01] and RM-AP22, 5.4 ± 0.4 mmHg [*p* < 0.01]) (Fig. 6).
- Comparisons of RM and RI models

In comparison with the RI model, the regurgitation rate was larger in the RM model, and the leakage rate was significantly larger in the RM and RM-AP22 models (Fig. 5a, b) (regurgitation: RI, 0.34 ± 0.02 L/min vs. RM, 0.48 ± 0.04 L/min [*p* < 0.05]; leakage: RI, 0.14 ± 0.01 L/min vs. RM, 0.28 ± 0.02 L/min [*p* < 0.01] and RM-AP22, 0.23 ± 0.01 L/min [*p* < 0.01]). The backflow rate of the RM model was the largest and differed significantly from that of the RI model (backflow rate: RI, 8.56% ± 0.38% vs. RM, 12.64% ± 0.79% [*p* < 0.01]) (Fig. 5c). ESA in the RM model reduced the regurgitation and backflow rate to levels comparable to those in the RI model. The forward flow rate in the RM model was larger than that in the RI model (RM, 6.05 ± 0.08 L/min vs. RI, 5.57 ± 0.08 L/min [*p* < 0.01]). The forward flow rate in the RM-AP22 was numerically larger than that in the RI but did not show a significant difference (RI, 5.57 ± 0.08 L/min vs. RM-AP22, 5.82 ± 0.09 L/min; *p* = 0.24), and the forward flow rate of the RI and RM-AP18 models were comparable (RM-AP18, 5.53 ± 0.11 L/min [*p* = 0.99]) (Fig. 5d).

The RI and RM-AP22 models showed comparable p-PG and m-PG values. In comparison with the RI model, the RM model showed a significantly lower p-PG value, and the RM-AP18 model showed a significantly higher m-PG value (p-PG: RI, 11.2 ± 0.6 mmHg vs. RM, 5.1 ± 1.3 mmHg [*p* < 0.01]; m-PG: RI, 6.2 ± 0.5 mmHg vs. RM-AP18, 9.5 ± 1.2 mmHg [*p* < 0.05]) (Fig. 6).

The comparisons of hemodynamic parameters between the two control groups are shown in Supplementary Table 1.

### Valve motion: leaflet-closing times

**Fig. 7:**
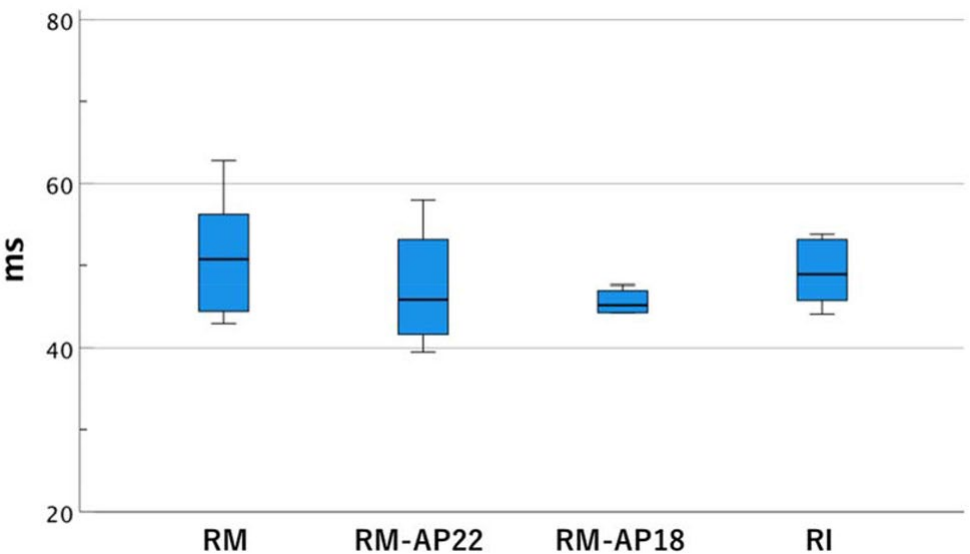
The comparison of valve-closing times. Valve-closing time was assessed with a high-speed camera. *P < 0.05; **P < 0.01

No significant differences were observed in the leaflet-closing time among the RM models with/without ESA and the RI models (Fig. 7).

### Root configuration

**Fig. 8:**
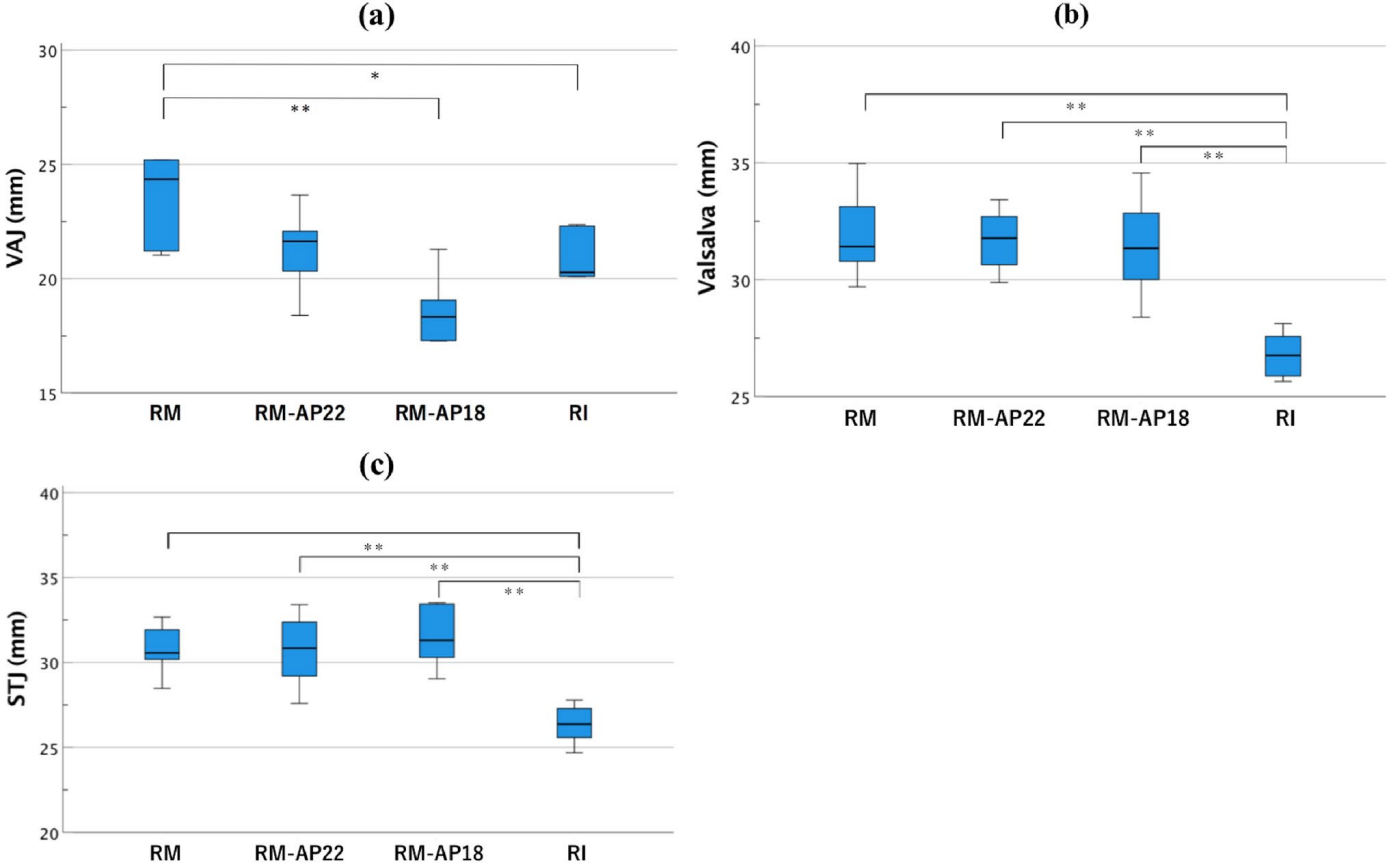
The comparison of aortic root configurations. The diameters were calculated from the cross-sectional perimeters of three areas. (**a**) VAJ diameter; (**b**) sinus of Valsalva diameter; (**c**) STJ diameter. *P < 0.05; **P < 0.01

Among the RM groups, the diameter of the VAJ in RM-AP18 was significantly smaller than the RM models without ESA (VAJ diameter: RM, 23.55 ± 0.79 mm vs. RM-AP18, 18.60 ± 0.61 mm [*p* < 0.01]; Fig. 8). No significant difference was observed in the diameters of the Valsalva and STJ in the RM group. The diameter of the VAJ in the RM model was larger than that in the RI model (VAJ: RM, 23.55 ± 0.79 mm vs. RI, 20.32 ± 0.86 mm [*p* < 0.05]; Fig. 8). These data suggest a lower annulus stability of the RM alone than the RI. The Valsalva diameter of the RI model was significantly smaller than those of the RM models, regardless of the addition of ESA (Valsalva diameter: RI, 26.79 ± 0.39 mm vs. RM, 31.90 ± 0.77 mm [*p* < 0.01]; RM-AP22, 31.70 ± 0.53 mm [*p* < 0.01]; and RM-AP18, 31.42 ± 0.90 mm [*p* < 0.01]). The diameter of the STJ in the RI model was also significantly smaller than those in the RM models (STJ diameter: RI, 26.35 ± 0.46 mm vs. RM, 30.73 ± 0.60 mm [*p* < 0.01]; RM-AP22, 30.71 ± 0.86 mm [*p* < 0.01]; and RM-AP18, 31.48 ± 0.72 mm [*p* < 0.01]) (Fig. 8).

The comparisons of the root configuration between the two control groups are shown in Supplementary Table 1.

**Fig. 9:**
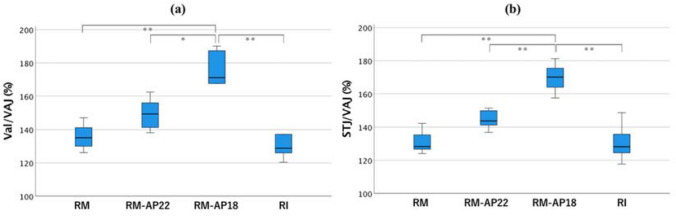
Valsalva/VAJ and STJ/VAJ ratio. Each ratio was calculated from the data examined using micro-CT. (**a**) Valsalva/VAJ ratio; (**b**) STJ/VAJ ratio. *P < 0.05; **P < 0.01

In the present study, the Valsalva/VAJ ratios were 135.82% ± 0.03% for RM, 149.48% ± 0.37% for RM-AP22, 170.13% ± 0.83% for RM-AP18, and 132.86% ± 0.05% for RI. The STJ/VAJ ratios were 130.85% ± 0.03% for RM, 144.50% ± 0.02% for RM-AP22, 169.70% ± 0.08% for RM-AP18, and 130.52% ± 0.04% for RI (Fig. 9).

## Discussion

This experimental study using a pulsatile flow simulator revealed that regurgitation, leakage, and backflow rates in RM alone were larger than those in RI. The addition of ESA to RM was effective for keeping the regurgitation and backflow rates comparable to those in RI. These findings suggest that RM with ESA with an adequate diameter and RI with neo-sinuses are comparable in terms of hemodynamics.

In this study, the hydrodynamic performances of the two modern VSRR techniques were quantitatively compared using a pulsatile circulation system. RM with annuloplasty and RI with neo-sinuses are considered similar in terms of structural features. Both techniques share the concept of reconstructing the sinus of Valsalva and aortic annulus stabilization. Recreation of the sinuses results in nearly normal aortic root behavior [6, 12]. Annulus stability is considered mandatory for avoiding recurrent aortic regurgitation (AR) [15].

In our pulsatile flow study, the RM valve without ESA showed less regurgitation control and annulus stability than the RI valve with sinuses. These findings are consistent with those reported by Maselli and Marom [16, 17]. Maselli reported effective height and coaptation height reduction with the RM technique in comparison with RI in the same aortic root [16]. Marom also reported that an increased aortic annular dimension was associated with effective height and coaptation height reduction [17]. Our micro-CT analysis revealed that the VAJ dimension was larger for RM than for RI, which was assumed to be associated with effective height reduction and the resultant increase in regurgitant flow in RM compared with RI. Annulus instability was obvious for the RM technique without any annuloplasty procedures.

Previous reports have indicated that the presence of the sinus of Valsalva decreases the stress acting on the valve leaflets, provides an effective orifice area, reduces the PG, and induces physiological and smooth valve motion [6, 12, 13, 18]. The ideal root configuration has been reported to correspond to a Valsalva/VAJ × 100 ratio of approximately 140–150% and STJ/VAJ × 100 ratio of 110–120% to remain within the physiological range [19, 20]. In the present study, RI showed the least expansion of the sinus of Valsalva, whereas RM-AP18 showed excessive expansion of the sinus of Valsalva and STJ relative to the VAJ diameter. In combination with the PG data, the findings suggested that ESA with a diameter of 18 mm to the 24-mm tube graft induced excessive tapering toward the annulus. We were surprised to see that a straight tube graft expanded more than the Valsalva graft with neo-sinuses. The bulge of the Valsalva is created in the RM, whereas RI mainly depends on the graft itself. In addition, we assume that the preserved interleaflet triangles in the RM led to a larger Valsalva diameter. The STJ/VAJ ratio increased beyond the ideal percentage for all VSRR models (ranging between 130.52 and 169.70%). In addition, STJ expanded significantly in the RM models compared to the RI. Thus, restriction of the STJ diameter may be required in addition to annular reduction, especially in the RM groups, when choosing a tube graft to achieve an ideal STJ/VAJ ratio. However, the correlation between the STJ/VAJ and valve configuration is another issue to be discussed. The need for STJ restriction could not be affirmed through our study alone.

Our study showed no marked differences in the valve-closing time between all RM and RI models. The presence of sinuses in all VSRR models may have contributed to a similar valve motion between the RM and RI models. The valve-moving velocity might have differed if the total cusp-moving distance had changed after the annuloplasty procedure. However, because of the limited visibility caused by the presence of VSRR grafts, the total cusp-moving distance could not be measured in this study.

Current clinical data suggest that careful patient selection and preservation of normal cusp geometry are essential for successful VSRR. Our study indicates that RM with annuloplasty and RI with neo-sinuses are comparable in terms of hemodynamics, presenting no superiority over the other.

This study had several limitations. First, there are anatomical differences between the porcine and human aortic roots, especially in the basal ring and VAJ. Muscle protrusion into the LVOT under the right coronary sinus is not frequently observed in the human anatomy. These anatomical differences may have affected the annuloplasty procedure and root structure. Second, we used a normal porcine aortic root without aortic annulus dilation. In addition, configurations of the cusps (such as effective height) could not be evaluated due to poor visibility through echocardiography. Thus, factors for durability and recurrent AR could not be evaluated. Third, ligation of the coronary ostia may have affected the valve motion. Fourth, grafts with sinuses used in the RI model were handcrafted because of limited availability. Thus, the geometries and dilation of the RI model might differ from those using a commercially available product. Nevertheless, the experimental methodology presented here is expected to be useful for investigating the optimal VSRR.

In conclusion, our experiments quantitatively clarified that RM alone was not sufficient to control regurgitant flow in comparison with RI. The addition of ESA to RM contributed to the reduction of regurgitation. However, an extensive reduction in diameter increased the transvalvular PG. The valve-closing time was comparable between the RM and RI techniques. The analysis using a micro-CT revealed a larger dilation of the sinus of Valsalva in the RM groups than in the RI group. RM with ESA with an adequate diameter and RI with neo-sinuses were considered comparable in terms of hemodynamics.

## Supplementary Information

Below is the link to the electronic supplementary material.

Supplementary file1 (DOCX 15 KB)

## Data Availability

The data in this article will be shared on reasonable request to the corresponding author.
