# Supplementary material for: Comparison of hemodynamics and root configurations between remodeling and reimplantation methods for valve-sparing aortic root replacement: a pulsatile flow study
Source: Surg Today. 2022 Nov 27;53(7):845–54. doi: 10.1007/s00595-022-02622-4 (PMC10290965; doi:10.1007/s00595-022-02622-4)
Supplement: Supplementary file 1 — Supplementary file1 (DOCX 15 KB) [file 595_2022_2622_MOESM1_ESM.docx]

|  | group | average | standard  deviation | p value |
| --- | --- | --- | --- | --- |
| Forward flow (L/min) | RM-C | 5.97 | 0.07 | 0.18 |
|  | RI-C | 5.88 | 0.14 |  |
| Regurgitation (L/min) | RM-C | 0.56 | 0.01 | 0.13 |
|  | RI-C | 0.51 | 0.08 |  |
| leakage (L/min) | RM-C | 0.34 | 0.06 | 0.61 |
|  | RI-C | 0.32 | 0.07 |  |
| Back flow rate (%) | RM-C | 15.17 | 0.80 | 0.13 |
|  | RI-C | 13.80 | 1.87 |  |
| peak PG (mmHg) | RM-C | 6.03 | 2.19 | 0.92 |
|  | RI-C | 6.13 | 1.11 |  |
| mean PG (mmHg) | RM-C | 3.75 | 1.52 | 0.93 |
|  | RI-C | 3.81 | 0.66 |  |
| VAJ (mm) | RM-C | 23.88 | 0.89 | 0.82 |
|  | RI-C | 23.67 | 1.96 |  |
| Val (mm) | RM-C | 35.19 | 2.64 | 0.76 |
|  | RI-C | 34.60 | 3.80 |  |
| STJ (mm) | RM-C | 27.95 | 2.47 | 0.56 |
|  | RI-C | 26.78 | 4.04 |  |
